# Supplementary material for: Coherent electron displacement for quantum information processing using attosecond single cycle pulses
Source: Sci Rep. 2020 Dec 14;10:21869. doi: 10.1038/s41598-020-79004-8 (PMC7736361; doi:10.1038/s41598-020-79004-8)
Supplement: Supplementary file 1 — Supplementary Information. [file 41598_2020_79004_MOESM1_ESM.pdf]

Supplementary Information for "Coherent electron displacement for quantum information processing using attosecond single cycle pulses"

Hicham Agueny

E-mail: hicham.agueny@uib.no

This PDF file includes:

Supplementary text

Figures S1 to S7

SI References

Supplementary Information Text

Coherent mixture of two states: Here, we provide further details about the spatiotemporal evolution of the electron density oscillating between two Rydberg states, where the mixture of the these two states, which defines a superposition state, is probed by a single-cycle pulse. The superposition state $|\psi\rangle$ considered in the manuscript is supposed to be prepared by a pump pulse in coherent mixture of two Rydberg state $|\chi_{n=9,l=8}\rangle$ and $|\chi_{n=10,l=9}\rangle$. Here, each state can be seen as a point defined by the polar angle θ_R on the Bloch sphere (see Fig. S1), which is a geometrical representation of the qubit state in space. We note that the change of a quantum state between two antipodes on the Bloch sphere can be done using a π-pulse excitation [1, 2]. In the manuscript, the time-dependent Schrödinger equation is solved for a fixed initial phase and polar angle.

The spatiotemporal evolution of the electron density at the end of the single-cycle pulse is shown in Fig. S2 for the polar angle $\theta_R = \pi/4$ rad and at several set of different initial phases (ϕ=0, $\pi/3$, $\pi/4$, $\pi/6$ and $\pi/2$ rad). The set of plots in Fig. S2 is equivalent of visualizing the spatial distribution of the electron density in the femtosecond time-scale (i.e. the values of τ are respectively, 0, 10.8, 16.2, 21.6 and 32.4 fs. We also show the real and imaginary parts of the initial and the final electron wavefunctions (see Fig. S3). This is displayed for an example of $\phi = \theta_R = \pi/4$. The corresponding

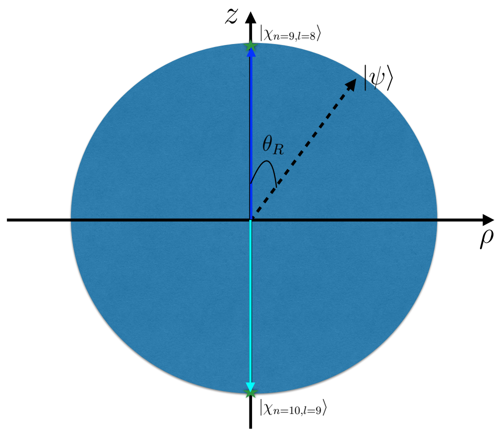

Figure S 1. Representation of the superposition state $|\psi\rangle$ on the Bloch sphere. The markers correspond to the two Rydberg states $|\chi_{n=9,l=8}\rangle$ and $|\chi_{n=10,l=9}\rangle$. θ_R is the polar angle. Due to the cylindrical symmetry, only the polar coordinate is viewed.

momentum distribution is presented in Fig. S4 in the case the electron dynamics is initiated from a single quantum state as well as the case of a coherent superposition of states. Specifically, in the first case $\theta_R = \pi/2$, while in the second case $\theta_R = \pi/4$ (see Eq. (1) in the main text). The results show a clear asymmetric distribution when the initial state is a mixture of two states (green curve). The asymmetry is the imprint of the interference between partial wave having opposite-parity (i.e. $l = 9$ and $l = 8$), while the distribution in the case of a single quantum state looks symmetric (black curve).

In order to illustrate the importance of the choice of the frequency of the single-cycle pulse to easily to retrieve the initial phase information over long distances, we calculate the expectation value of the electron momentum $< p_z >$ induced by the field at the frequency ω =0.0006 a.u. Note that this frequency is too small to undergo a complete displacement of the electron wavepacket, as discussed in the main text (see Fig. 2(a)). The result is presented in Fig. S5 (black curve) at the peak intensity of 4.78 10^{10} W/cm^2. It is seen that the $< p_z >$, although it oscillates, it exhibits an asymmetric behavior. And Because of this asymmetric profile, it cannot be fitted precisely and properly to the symmetric function described in Eq. 3 in the mains text, also shown in Fig. S5 (blue curve), and hence comes the difficulty for retrieving the initial phase information. For reference, the $< p_z >$ in the absence of the single-cycle field is shown (green curve).

Entangled electron-electron state: An extension to a two-electron system is shown in Fig. 6S for a prototype of a Heluim atom. Here calculations are based on a one-dimensional (1D) model and where the correlated electron-electron interaction is taken into account. Details about the calculations are described in the main manuscript. We use a single-cycle pulse with a peak intensity of 8 10^{22} W/cm^2 and an angular frequency of 5.51 a.u. (the corresponding duration is 13 as). At the end of the probing pulse, the results of observing the entangled electron-electron state far away from the nucleus are

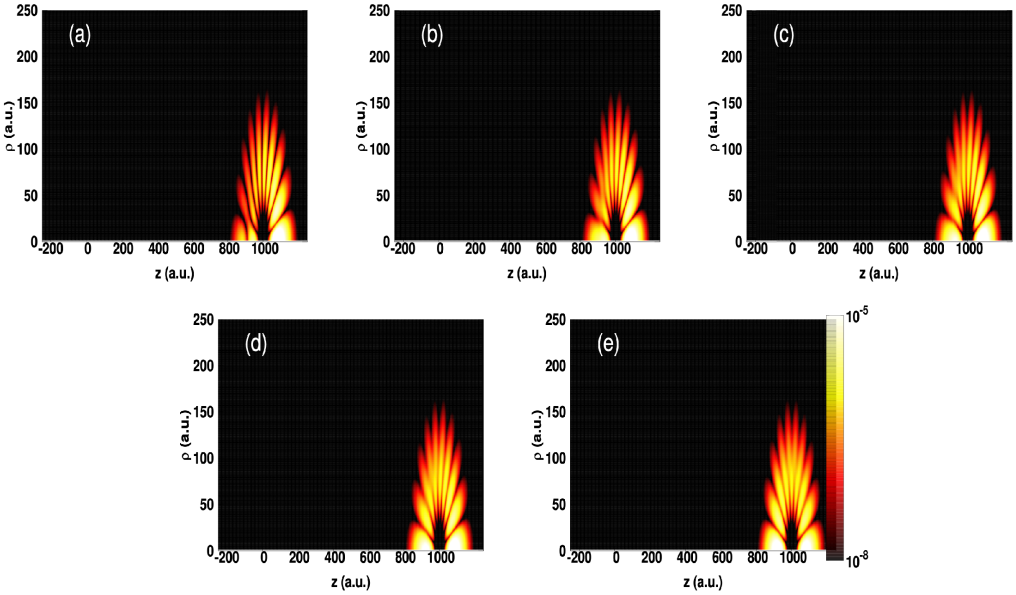

Figure S 2. Electron density in z-ρ plane at the end of the probing single-cycle pulse at the polar angles $\theta_R = \pi/4$ rad and for various initial phases ϕ: (a) $\phi = 0$; (b) $\phi = \pi/6$; (c) $\phi = \pi/4$; (d) $\phi = \pi/3$; (e) $\phi = \pi/2$ rad. The peak intensity is 4.26 10^{18} W/cm^2 and the angular frequency is 0.057 a.u..

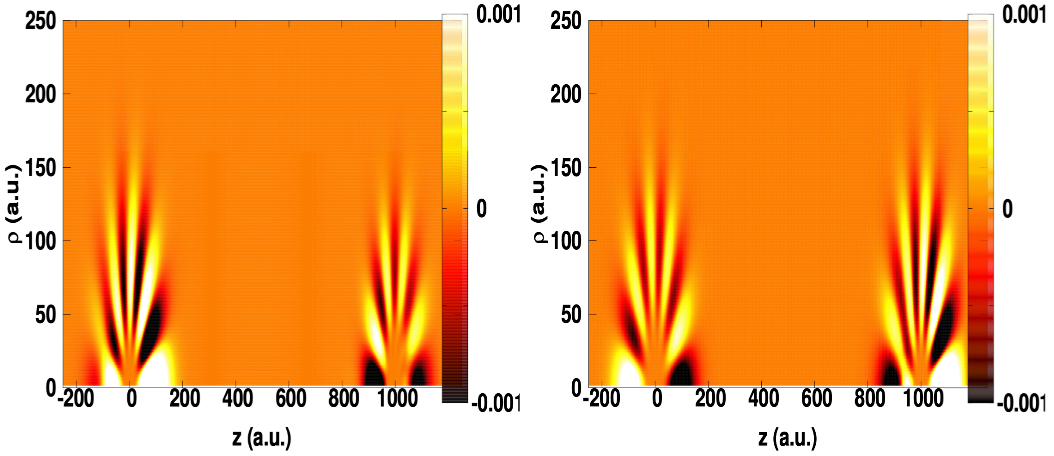

Figure S 3. Electron wave function of a superposition state in z-ρ plane at the end of the probing single-cycle pulse for the polar angle $\theta_R = \pi/4$ and the initial phase $\phi = \pi/4$ rad. (left-hand side) real part and (right-hand side) imaginary part of the wave function. The peak intensity is 4.26 10^{18} W/cm^2 and the angular frequency is 0.057 a.u.. For reference, the initial state wave functions before introducing the probing pulse are shown at the origin.

shown in Fig. 6S for both the ground state (cf. Fig. 6S(a)) and the first singlet state (cf. Fig. 6S(b)). Here, the initial states are exact and stemming from the diagonalization of the Hamiltonian, which contains also the correlated interaction, unlike those obtained in the main manuscript (see. Eq. 4 in the main manuscript), which are built-up based on one-electron eigenstates. Also is shown for reference the density of the initial state

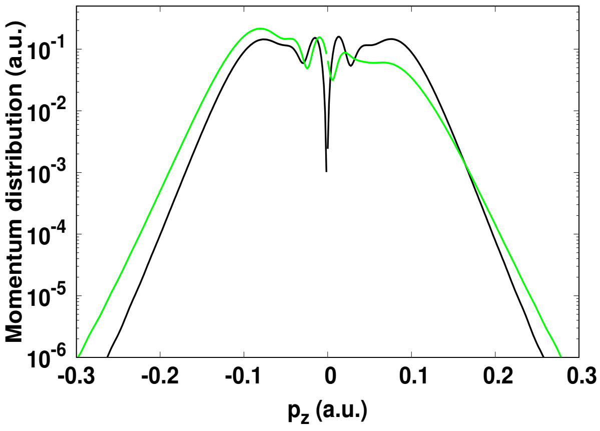

Figure S 4. Momentum distribution integrated over the momentum along the ρ-direction in the case of a single quantum state (i.e. $\theta_R = \pi/2$) (black curve) and in the case of a coherent superposition of state (i.e. $\theta_R = \pi/4$) (green curve). The initial phase information is $\phi(t_i) = \pi/4$. The peak intensity is 4.26 10^{18} W/cm^2 and the angular frequency is 0.057 a.u..

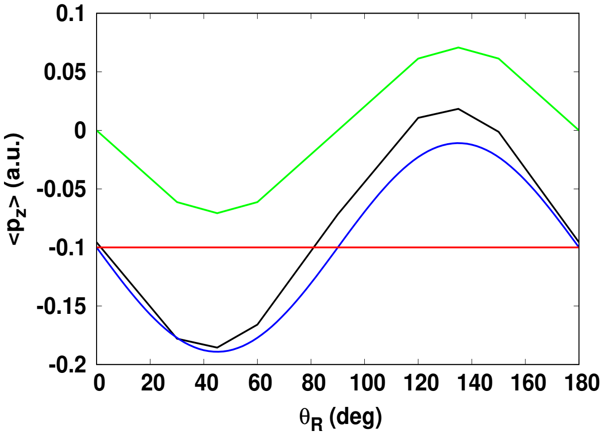

Figure S 5. Expectation value of the momentum $< p_z >$ as a function of the polar angle θ_R at the initial phase $\phi = \pi/4$ in the presence of the single-cycle pulse (black solid line) and in the absence case (green line). The peak intensity is fixed at 4.78 10^{10} W/cm^2 and angular frequency is 0.0006 a.u.. Also is shown the data from the simple model $-0.1 + 0.1\cos(0.9\pi + \pi/4)\sin(2\theta_R)$ (blue line). The red line is for guiding.

which is localised at the origin. It can be seen that the transfer of the ground state results in a significant change of the wavefunction with respect to the excited state. However, increasing the angular frequency up to 18.37 a.u. (the corresponding duration is 4.1 as) and the peak intensity to 10^{25} W/cm^2 allows one to preserve the nature of the correlated electron wavepacket, as shown in Fig. 6S(c). Here, relativistic single-cycle pulses [3] become necessary to explore this range of the peak intensity, which could enable us to explore phenomena occurring on the zeptosecond (10^{-21} s) time scale.

Non-dipole effects on electron displacement:

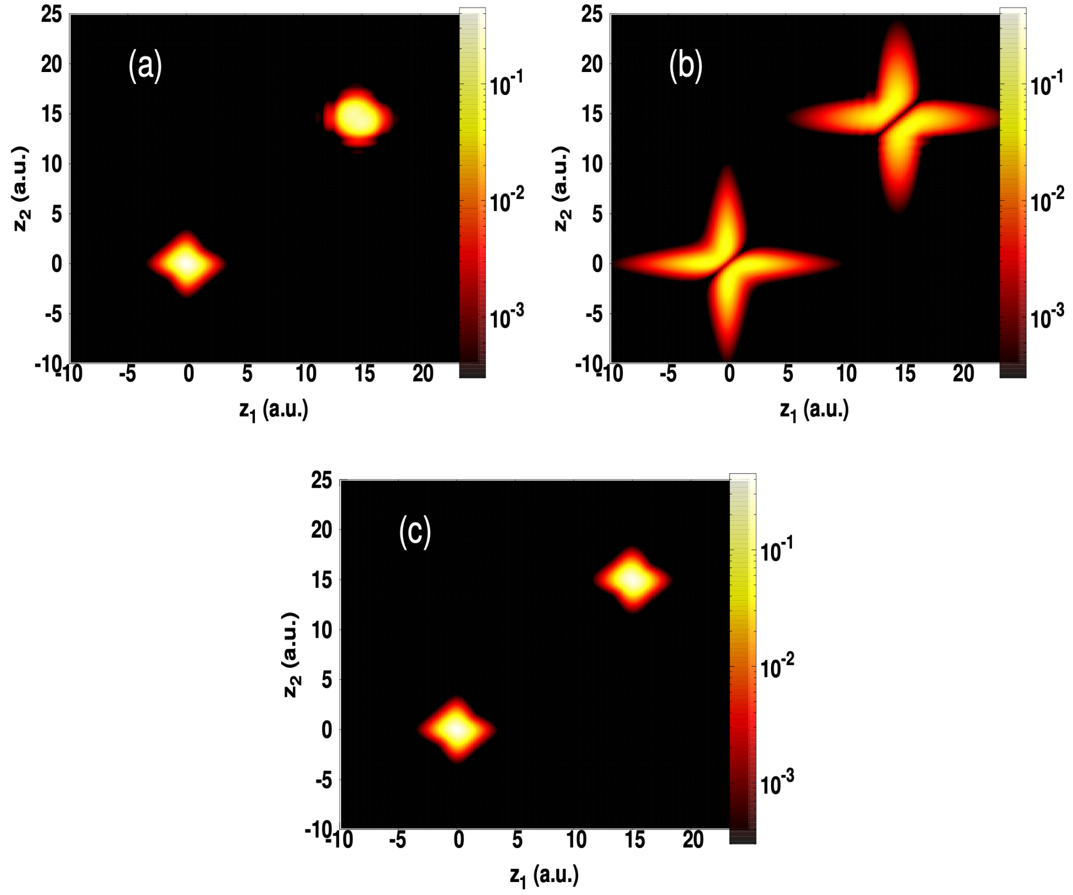

Figure S 6. (a)-(b): Electron density in z-ρ plane at the end of the probing single-cycle pulse at the polar angles $\theta_R = \pi/4$ rad and for various initial phases ϕ: (a) $\phi = 0$; (b) $\phi = \pi/6$; (c) $\phi = \pi/4$; (d) $\phi = \pi/3$; (e) $\phi = \pi/2$ rad. The peak intensity is 4.26 10^{18} W/cm^2 and the angular frequency is 0.057 a.u.. For reference, the electron density before introducing the probing pulse is shown in (d)

The calculations performed in the present work are done under the dipole approximation. To investigate how non-dipole effects might modify our results obtained, we perform additional calculations using a 2D-model, in which non-dipole effects are taken into account. We use a similar model as the one in [4]. The Hamiltonian of the 2D system (i.e atom + laser field) has the form

$$H(t_n) = -\frac{\mathbf{p_x}^2}{2} - \frac{\mathbf{p}_z^2}{2} - \frac{1}{\sqrt{x^2 + z^2 + 0.64}} + \mathbf{A(t_n)} \cdot \mathbf{p}_z + \frac{\mathbf{A(t_n)}^2}{2}, \qquad (1)$$

where the retardation $t_n = t - x/c$ is included in the vector potential $A(t)$; c is speed of light. The laser field is considered to be linearly polarized along the z-axis, and is propagating along the x-axis. The results beyond dipole approximation are summarized in Fig. S6 for an example of an electron wavepacket initially prepared in the $3p_z$ state of hydrogen atom. The calculations are performed for a peak intensity of 4 10^{20} W/cm^2 and an angular frequency of 27.2116 a.u. Here the final position of the electron wavepacket is expected to be at $\alpha(t_f) = 1.178 U_p$ =31.4 a.u.. In Fig. S7(a) it is seen that the

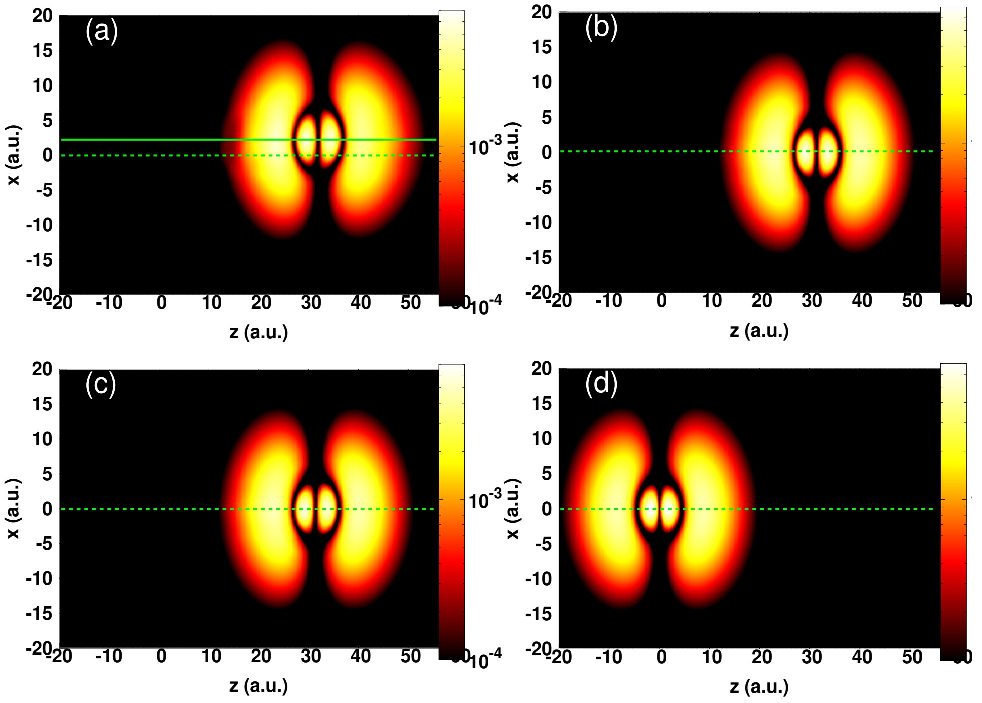

Figure S 7. Electron density in z-x plane at the end of the probing single-cycle pulse for an initial state $3p_z$. Calculations are performed beyond dipole approximation (a), within dipole approximation (b) and when neglecting the diamagnetic term ($\mathbf{A}^2$). The peak intensity is 4 10^{20} W/cm^2 and the angular frequency is 27.2116 a.u.. For reference, the electron density before introducing the probing pulse is shown in (d). The green line is for identifying the spatial shift due to the magnetic field. The green dashed line passed from p_x =0 is plotted for reference.

electron wavepacket is slightly shifted along the propagation direction (i.e x-axis). For reference, the results obtained within the dipole approximation are shown in Fig. S7(b). To understand the origin of the observed small spatial shift, we show in Fig. S7(c), calculations including only non-dipole effects via the term $\mathbf{A} \cdot \mathbf{p}$ in the Hamiltonian (i.e. the diamagnetic term $\mathbf{A}^2$ [5] is neglected). The results remain unchanged. Here, although the magnetic field induces a transverse spatial shift, it does not alter the shape of the electron wavepacket, which is an important condition for preserving a transferred information.

[1] T. H. Stievater, X. Li, D. G. Steel, D. Gammon, D. S. Katzer, D. Park, C. Piermarocchi, and L. J. Sham, Rabi oscillations of excitons in single quantum dots. Phys. Rev. Lett. **87**, 133603 (2001).

[2] A. Zrenner, E. Beham, S. Stufler, F. Findeis, M. Bichler, and G. Abstreiter, Coherent properties of a two-level system based on a quantum-dot photodiode. Nature **418**, 612-214 (2002).

[3] Z. Nie, C-H. Pai, J. Hua, C. Zhang, Y. Wu, Y. Wan, F. Li, J. Zhang, Z. Cheng, Q. Su *et al.*, Relativistic single-cycle tunable infrared pulses generated from a tailored plasma density structure, Nat. Photonics **12**, 489-494 (2018).

[4] S. Chelkowski, A. D. Bandrauk, and P. B. Corkum, Photon-momentum transfer in multiphoton ionization and in time-resolved holography with photoelectrons. Phys. Rev. A **92**, 051401 (2015).

[5] G. A. Aucar, T. Saue, L. Visscher, and H. J. Aa. Jensen, On the origin and contribution of the diamagnetic term in four-component relativistic calculations of magnetic properties J. Chem. Phys. **110**, 6208 (1999).
